# Roles of hormones in regulating root growth–water interactions

**DOI:** 10.1093/jxb/eraf063

**Published:** 2025-02-13

**Authors:** Shivam Sharma, Malcolm J Bennett, Poonam Mehra

**Affiliations:** Department of Plant Molecular Biology, University of Delhi South Campus, New Delhi 110021, India; Plant and Crop Science, School of Biosciences, University of Nottingham, Sutton Bonington Campus, Leicestershire LE12 5RD, UK; Plant and Crop Science, School of Biosciences, University of Nottingham, Sutton Bonington Campus, Leicestershire LE12 5RD, UK; Pennsylvania State University, USA

**Keywords:** Hydropatterning, hydrotropism, plant hormones, root adaptive responses, root branching, water stress, xerobranching

## Abstract

Water stress presents a critical challenge affecting plant growth and agricultural productivity, with drought alone causing substantial yield losses. Roots serve as the primary site for water uptake, enabling plants to detect water stress by sensing changes in soil moisture levels. This initial perception prompts roots to initiate a spectrum of adaptive responses at morphological, anatomical, and biochemical levels. In addition to coping with severe water stress conditions such as drought, roots also respond to microscale variations in water availability within the rhizosphere as they navigate through soil, exhibiting responses such as hydrotropism, xerobranching, and hydropatterning. These adaptive responses are orchestrated by dynamic and sophisticated sensing and signalling mechanisms mediated by plant hormones at the cellular level. This review explores recent advances in our understanding of root responses to water stress, emphasizing the hormonal mechanisms underpinning these adaptations. Furthermore, it outlines future perspectives aimed at enhancing crop resilience to water stress through improved understanding and manipulation of root–water interactions.

## Teaser

This review highlights recent advances in understanding hormone-mediated mechanisms underlying root responses to water stress.

## Introduction

Securing access to water is vital for all living organisms, particularly plants which rely on their roots for uptake. Climate change has intensified water stress, leading to significant global agricultural losses estimated at US$30 billion over the past decade (Gupta *et al*., 2020). Plant roots, serving as primary sensors and responders to water deficits, play a crucial role in mitigating water stress and maintaining vital functions. In response to changes in water availability, roots exhibit remarkable plasticity by adjusting their architecture to maximize water uptake from the soil (Karlova *et al*., 2021; Voothuluru *et al*., 2024). This plasticity is extremely important given the increasing frequency and severity of droughts driven by climate change.

Plant hormones are recognized as key regulators in shaping how roots respond to fluctuations in water availability. These compounds orchestrate signalling pathways that regulate root growth and development under stressful conditions (Waadt *et al*., 2022). For instance, recent studies have revealed a complex ‘water-sensing’ mechanism where hormone dynamics interact with soil hydrodynamics to finely modulate root branching patterns (Mehra *et al*., 2022). Understanding these molecular interactions provides critical insights into enhancing crop water use efficiency.

**Fig. 1.**
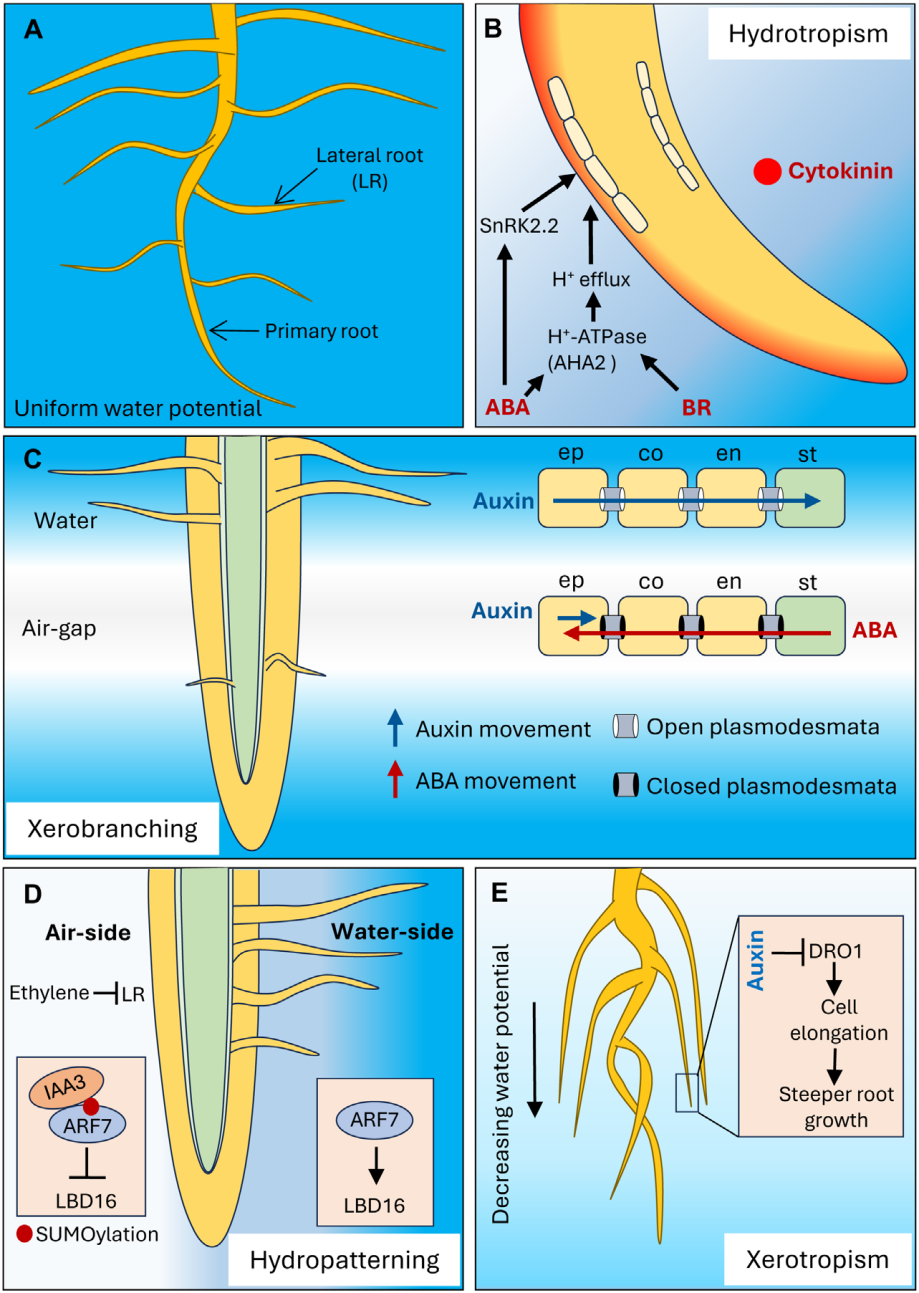
Hormones mediate root responses to water availability. (A) Under homogeneous water distribution in soil, lateral roots (LRs) develop symmetrically, resulting in a uniform root system. (B) Root tips show hydrotropism and bend towards areas with high water potential. ABA drives asymmetric expansion of cortical cells on the convex side of roots facing low water potential by the action of SnRK2.2. Both ABA and BR promote hydrotropism via the activity of the proton pump AHA2. Cytokinin (in red) exhibits asymmetric distribution at the root tip to regulate hydrotropism. (C) Growing root tips pause branching in air gaps (xerobranching). Lack of moisture triggers ABA release from the stele (st) to the outer root tissues, blocking the radial inward symplastic movement of auxin to pause root branching. ABA-mediated repression is relieved in the presence of water. ep, epidermis; co, cortex; en, endodermis. (D) Asymmetric SUMOylation of the auxin response factor ARF7 induces asymmetric distribution of lateral roots, leading to hydropatterning. Ethylene suppresses LR formation on the air side of the root. (E) Soil drying decreases water potential vertically, promoting steeper root growth or xerotropism by the action of *Deeper Rooting 1* (*DRO1*). Auxin negatively regulates *DRO1*, triggering downward bending. Water availability is represented as a blue colour gradient.

This review consolidates recent knowledge on the critical role of hormones in mediating root–water interactions, emphasizing recent advances in understanding how hormones regulate root responses to different water conditions, including variations in water availability, drought, and flooding (Fig. 1A–E). Exploring these pathways is relevant for developing strategies to breed resilient crops that can withstand fluctuations in water availability.

## Roots responses to heterogenous water availability in soil

### Hydrotropism

Hydrotropism is characterized by directional bending of root tips towards soil patches with higher water availability (Fig. 1B). Multiple studies highlight the critical role of abscisic acid (ABA) in regulating this response, as ABA biosynthetic and signalling mutants are impaired in hydrotropic bending. Specifically, the protein kinase SnRK2.2, a positive regulator of ABA signalling, acts asymmetrically in the root elongation zone to promote cortical cell expansion on the convex side of the root facing low water potential (Dietrich *et al*., 2017). This asymmetric ABA response in the elongation zone of hydrostimulated roots drives hydrotropic bending of root tips towards regions with higher water availability (Fig. 1B). Recently, it was discovered that low concentrations of ABA promote hydrotropism through increased activity of H^+^-ATPase2 (AHA2) which facilitates proton extrusion in the apoplast (Miao *et al*., 2021). Similarly, brassinosteroid (BR) receptor (BRI1) also interacts with AHA2 to promote hydrotropism (Miao *et al*., 2018). However, the precise mechanism by which AHA2 regulates hydrotropism remains unclear and requires further investigation. Moreover, vasculature-derived BR signalling has been shown to promote hydrotropism, as loss-of-function mutants of phloem-localized BRI1-like receptor (BRL3) exhibits an impaired hydrotropic response (Fàbregas *et al*., 2018; Gupta *et al*., 2023, Preprint). Further research is necessary to elucidate how phloem-derived BR signalling regulates the asymmetric expansion of cortical cells during hydrotropism.

While asymmetric cell expansion in hydrostimulated roots has been observed, it remains uncertain if this response is preceded by an asymmetric distribution of hormones in the elongation zone. Among phytohormones, cytokinin is noted for exhibiting asymmetric distribution during hydrotropism. Cytokinin triggers asymmetric cell division on the dry side of the root facing low water potential (Chang *et al*., 2019). Further investigations are needed to fully understand the specific role of cytokinin and its interplay with ABA in regulating hydrotropism. Concerning another phytohormone, auxin, studies present conflicting results about its asymmetrical distribution preceding hydrotropic bending. Shkolnik *et al*. (2016) reported no asymmetrical distribution of indole-3-acetic acid (IAA) prior to bending in Arabidopsis. In contrast, Wang *et al*. (2020) demonstrated asymmetrical auxin redistribution, with higher auxin levels on the dry side of maize roots. The authors suggested that this asymmetry in auxin levels could promote increased cell expansion, thereby contributing to hydrotropic bending. Additionally, the authors emphasized that hydrotropic bending in crop species such as maize requires not only asymmetric cell expansion but also asymmetric cell division/flux, coordinated by multiple hormones. Importantly, Wang *et al*. (2020) provided a more comprehensive analysis of hormones in hydrostimulated roots, highlighting additional key players in hydrotropism. Besides auxin, the authors observed increased levels of ABA on both dry and wet sides of hydrostimulated maize roots, while salicylic acid (SA) and zeatin showed differential accumulation. Higher SA levels were detected on the wet side, which may suppress cell elongation to drive bending. Interestingly, the increased zeatin levels on the wet side contrast with findings in Arabidopsis, suggesting potential species-specific differences in cytokinin’s role in hydrotropism that warrant further investigation.

To validate these findings, real-time monitoring of hormone dynamics in hydrostimulated roots would be valuable. The application of hormone biosensors in crop species remains technically challenging, but such analyses, as demonstrated by Wang *et al*. (2020), provide a foundation for testable hypotheses that can be explored further in model systems such as Arabidopsis.

### Xerobranching

Roots often encounter large air spaces or gaps within soil, where they suppress lateral root formation. This adaptive response, known as xerobranching, is regulated by ABA (Orman-Ligeza *et al*., 2018). Recently, a ‘hydrosignalling’ mechanism underlying xerobranching has been proposed (Fig. 1C). Hydrodynamic modelling revealed that in the absence of external water, phloem-derived water becomes the primary source of water for growing root tips. When roots are exposed to air gaps, phloem water moves outward from the vascular tissues to sustain root growth. This reversal in water flux co-mobilizes ABA from its source (i.e. phloem companion cells), to the outer root tissues, where it triggers the closure of cytoplasmic bridges known as plasmodesmata. These events block the symplastic movement of auxin from the epidermis to the vascular tissues where lateral roots initiate. Notably, the transient closure of plasmodesmata is reversible. When roots regain access to water, ABA levels decrease, plasmodesmata reopen, and lateral root growth resumes (Mehra *et al*., 2022).

This hydrosignalling mechanism illustrates that hormones act as hydrosignals to initiate moisture-driven signalling. Hydrosignalling involves dynamic redistribution of hydrosignals such as auxin and ABA in response to heterogenous water availability. By integrating water fluxes with the redistribution of these hydrosignals, roots can adapt their branching patterns according to soil water availability.

### Hydropatterning

Soil water distribution also determines the radial arrangement or patterning of lateral roots, a phenomenon known as hydropatterning, where roots preferentially position their branches towards areas with higher water availability (Bao *et al*., 2014). Both hydropatterning and xerobranching optimize root architecture for efficient water uptake and resource acquisition. Unlike xerobranching, which is regulated by ABA, hydropatterning primarily relies on auxin signalling pathways (Bao *et al*., 2014; Mehra *et al*., 2022). Orosa-Puente *et al.* (2018) revealed that AUXIN RESPONSE FACTOR 7 (ARF7) undergoes post-translational modification in response to radial water gradients, leading to asymmetric initiation of lateral roots (Fig. 1D). Specifically, ARF7 is SUMOylated on the air-exposed side of the root. SUMOylated ARF7 preferentially recruits the auxin signalling repressor IAA3, which inhibits ARF7 activity and suppresses lateral root formation on the dry side of the root. Conversely, on the water-exposed root side, non-SUMOylated ARF7 continues to promote lateral root initiation via LATERAL ORGAN BOUNDARIES-DOMAIN 16 (LBD16). This mechanism highlights the important role of post-translational modifications in regulation of root responses to varying water availability in complex soil environments.

Recently, Scharwies *et al*. (2025) revealed significant phenotypic variability for hydropatterning in an association panel of maize inbred lines. This study revealed that a large portion of temperate maize germplasm showed weakened hydropatterning. Genome- and transcriptome-wide association studies (GWAS/TWAS) revealed that both auxin and ethylene signalling pathways are associated with hydropatterning response. While auxin promotes lateral root development on moisture-contacting root surfaces, ethylene suppresses root branching on air-exposed surfaces independently of auxin (Fig. 1D). This study showed that investigating genotypic diversity of microscale adaptations can provide deeper understanding of molecular pathways regulating moisture-responsive root growth.

The sensitivity of roots to such radial water gradients is also influenced by their growth rates. Faster-growing root tips generate stronger water potential gradients within their tips, promoting a more pronounced hydropatterning response (Robbins and Dinneny, 2018). This study suggests that plants perceive radial water gradients within a ‘competence zone’ at root tips, which also coincides with the site where auxin primes lateral root formation. However, further research is needed to fully understand how plants sense these gradients to shape their root architecture. Future studies should also explore whether hydropatterning involves the radial movement of auxin and hydraulic fluxes to fine-tune root responses to soil moisture variations.

Radial water gradients also impact the patterning of root hairs, which tend to emerge preferentially on the air-exposed side of roots (Bao *et al*., 2014). Duddek *et al*. (2024) recently used synchrotron-based X-ray μCT (micro computed tomography) to visualize root hair patterning in maize roots grown in soil. Their study revealed that root hairs predominantly grow in air-filled pores at the root–soil interface. While treatments with ABA or the ethylene precursor, 1-aminocyclopropane-1-carboxylic acid (ACC), have been shown to promote root hair initiation on the water-exposed side, the exact molecular mechanisms underlying root hair patterning remain elusive.

## Root responses to extreme water stress conditions

### Drought

Apart from the local responses to heterogenous water availability, roots undergo various changes to enhance water uptake and sustain plant growth under limited water supply. This includes alterations in root length, branching, number, angle, root diameter, biomass, lignification, and aerenchyma formation (Pandey and Bennett, 2019; Ranjan *et al*., 2022; Waadt *et al*., 2022; Zhang *et al.*, 2024). One notable response to soil drying is xerotropism, where roots grow deeper and steeper in search of water (Fig. 1E). Xerotropism involves lateral roots reorienting their growth direction from shallow to steeper angles in response to water deficit. To promote deeper rooting, roots enhance response to gravity. This response appears to be distinct from hydrotropism, as mutants defective in hydrotropism still show xerotropism (Feng *et al*., 2016).

Auxin plays a significant role in xerotropism. The auxin receptor mutant *tir1* does not reorient lateral roots under water deficit conditions (Rellán-Álvarez *et al*., 2016). Additionally, Uga *et al*. (2013) showed that the expression of the auxin-regulated gene *DRO1* (*Deeper Rooting 1*) is linked to steeper root growth in rice. Auxin negatively regulates *DRO1* on the lower side of gravistimulated roots, resulting in reduced cell elongation compared with the upper side, which causes the roots to curve downward (Uga *et al*., 2013). Recent studies in maize have revealed ABA-mediated induction of DRO1 in response to water deficit, highlighting the role of ABA in regulating root angle in response to soil water availability (Feng *et al*., 2022).

In addition to morphological changes, ABA also triggers anatomical changes and promotes xylem differentiation under water-limiting conditions to facilitate uninterrupted water movement (Ramachandran *et al.*, 2018, 2021; Bloch *et al.*, 2019). Furthermore, ABA (and auxin) also promotes the formation of drought-induced rhizosheaths—a root–soil interface consisting of root hairs and mucilage (Zhang *et al*., 2020; Karanja *et al*., 2021). This enhances water uptake efficiency by improving soil porosity and water retention under limited water supply. ABA also interacts antagonistically with cytokinin to fine-tune root responses to water stress (Huang *et al*., 2018).

While ABA’s role in regulating root responses to drought has been widely studied, the involvement of other phytohormones in root adaptation remains less explored and requires further attention. For instance, soil drying can elevate endogenous levels of jasmonic acid (JA) in roots (de Ollas *et al*., 2018; Castro-Valdecantos *et al*., 2021), but its precise effect on root adaptations is not fully understood. It is plausible that JA enhances water conductivity from soil into roots during water stress (Sánchez-Romera *et al.*, 2014). On the other hand, contradictory evidence suggests that low JA levels promote root length and branching, and thereby enhance adaptation to moderate soil drying (Dhakarey *et al.*, 2017). Additionally, recent studies by Gabay *et al*. (2023) have demonstrated that wheat plants with loss-of-function mutations in a JA biosynthetic gene (*12-Oxophytodienoate Reductase*; *OPRIII*) exhibit longer seminal roots, while increased *OPRIII* dosage or overexpression leads to reduced root growth. Gabay *et al*. (2021, 2023) revealed that the dosage of *OPRIII* plays a crucial role in regulating root growth and yield under drought conditions in wheat. These studies underscore the importance of JA biosynthesis in regulating root architecture and drought resilience, highlighting the need for further exploration of JA’s role in drought tolerance mechanisms.

### Flooding

In contrast to drought, waterlogged conditions necessitate shallow root growth to optimize gas exchange in hypoxic environments, where diffusion is limited. Cytokinin signalling mediates horizontal lateral root growth, acting as an anti-gravitropic signal (Waidmann *et al*., 2019). Further, to enhance tolerance to low-oxygen environments, roots exhibit adaptations such as formation of aerenchyma, emergence of aerenchymatous adventitious roots from stem nodes into soil or water, an increase in cortex-to-stele ratio to enhance oxygen diffusion, and the induction of a radial oxygen loss (ROL) barrier at basal zones to minimize oxygen loss (Pedersen *et al.*, 2021). Ethylene acts as the master regulator for most of these responses (Wang and Komatsu, 2022). For instance, ethylene accumulates under oxygen-deficient conditions and induces programmed cell death in cortical cells, promoting aerenchyma formation (Yamauchi *et al*., 2017). Similarly, auxin signalling pathways are also involved in constitutive aerenchyma formation (Yamauchi *et al.*, 2019). Further, ABA induces the formation of suberin lamellae in the exodermis, contributing to the ROL barrier formation in rice (Shiono *et al*., 2022). These hormonal interactions and adaptive strategies enable roots to survive and function under low-oxygen conditions, essential for plant resilience in flooded environments.

## Addressing key knowledge gaps in root adaptations to water stress

Given the challenges posed by climate change, studying root adaptive responses to water stress is of paramount importance. These responses not only elucidate the initial stages of stress perception but also offer valuable insights into conserved mechanisms across different types of water-related stress such as osmotic stress, salinity stress, and soil compaction. Recent advances in studies exploring root–water interactions (Box 1) have significantly increased our understanding and opened up several key questions for future research. Filling these knowledge gaps will be essential to engineer crops that can withstand varying water availability, ultimately contributing to sustainable agriculture and food security.

Box 1. **Key developments in hormonal regulation of root responses to water availability**

**ABA**

**• Asymmetric acidification of the apoplast promotes hydrotropism**

Miao *et al.* (2021) showed that a hydrotropic stimulus triggers asymmetric H^+^ extrusion on the convex side of the root at an early stage. This H^+^ extrusion across the plasma membrane is mediated by the proton pump, AHA2. Low concentrations of ABA promote the activity of AHA2 by relieving its inhibition mediated by PP2Cs. Acidification of the apoplast may promote cell wall loosening and facilitates cell elongation on the dry side of the root.

**• ABA regulates root growth angle in response to water deficit**

Feng *et al*. (2022) identified ABA response factor-binding elements in the promoter of maize *DRO1* (*Deeper Rooting 1*) facilitating ABA-mediated induction of *DRO1*. This mechanism enhances deeper rooting in response to water deficit conditions, improving the plant’s ability to access water.

**• Hydraulic flux-responsive hormone redistribution determines root branching**

Mehra *et al*. (2022) proposed the hydrosignalling model to explain how roots adjust their branching pattern in response to external water availability. Hydrosignals such as ABA co-mobilize with phloem-derived water when roots are transiently exposed to air gaps. ABA induces the closure of symplastic pathways (plasmodesmata), blocking the radial inward movement of another hydrosignal, auxin, from the outer root tissues to the lateral root stem cells. This redistribution of hormones suppresses root branching in air gaps, triggering the xerobranching response.

**Auxin**

**• Auxin redistribution precedes hydrotropic bending**

Wang *et al.* (2020) quantitated IAA levels in roots and revealed that IAA redistribution occurs following hydrostimulus in maize roots. This contrasts with previous findings in Arabidopsis, which suggested no auxin redistribution during hydrotropism (Shkolnik *et al*., 2016).

**Ethylene**

**• Ethylene acts on the air side of the root to regulate hydropatterning**

Scharwies *et al*. (2025) revealed significant phenotypic variability in hydropatterning among maize inbred lines. The authors showed that while auxin promotes lateral root growth on moisture-contacting surfaces, ethylene suppresses branching on air-exposed surfaces of the root.

**Brassinosteroid**

**• Phloem-derived brassinosteroid signalling is essential for root hydrotropism**

Fàbregas *et al*. (2018) showed that BRL3, a phloem-specific BR receptor, is essential for proper hydrotropic response, as the *brl3* mutant shows defective hydrotropic bending. However, the precise regulatory mechanisms still remain unclear.

**Jasmonic acid**

**• JA biosynthesis regulates root architecture and drought resilience**

Gabay *et al.* (2021, 2023) showed that the dosage of the JA biosynthetic gene *OPRIII* significantly influences seminal root growth and yield in wheat under water-limiting conditions. These findings emphasize JA’s potential as a target for improving drought tolerance mechanisms.

### How does phloem-derived hormone signalling regulate root adaptive responses?

Despite considerable research into ABA’s role under water stress, there remains ongoing debate about its origin within root tissues. Recent findings by Mehra *et al*. (2022) have provided new insights into the role of phloem in releasing ABA during transient water stress. With the help of a high-resolution ABA biosensor, the authors demonstrated ABA unloading from phloem in root tips exposed to air gap conditions. This study specifically pinpointed phloem companion cells as the primary source of ABA during water stress. This discovery aligns with earlier research (Kuromori *et al*., 2014) that localized ABA biosynthetic enzymes within phloem companion cells. Additional evidence suggests that vascular derived BR signalling also plays an important role in regulating hydrotropism (Gupta *et al*., 2023, Preprint). This further supports the significant role of phloem in coordinating root responses to water stress. Future studies should aim to elucidate the mechanistic basis of how these hormone signals, unloaded from phloem into root tips, influence downstream signalling in root tissues.

### What is the role of hormones and symplastic signalling in perceiving water stress?

Intercellular or symplastic communication via plasmodesmata plays a crucial role in regulating plant development and stress responses (Tee and Faulkner, 2024). Studies show significant association between plasmodesmata function and cellular turgor. For instance, a 200 kPa difference in cell turgor leads to a 50% reduction in plasmodesmata permeability (Oparka and Prior, 1992). Moreover, mechanosensing-mediated closure of plasmodesmata has been proposed (Park *et al*., 2019). These findings suggest that plasmodesmata may play a broader role in controlling root adaptive responses under fluctuating water availability.

Given that most plant hormones are small (<500 Da) and soluble, they can easily traffic through plasmodesmata. Recent evidence suggests trafficking of hormones such as auxin, BRs, and ABA through these channels (reviewed by Tee and Faulkner, 2024). Although studies have linked plasmodesmata closure to responses such as xerobranching (Mehra *et al*., 2022), there has been no direct real-time visualization of hormone movement through these channels. Additionally, further research is required to understand how turgor pressure within cells regulates the structure and function of plasmodesmata in response to fluctuating water availability. It is also not clear if plasmodesmata closure helps prevent symplastic water loss during limited water supply.

### Where is the water-sensing niche in root tips?

One important question that needs investigation in terms of root–water interactions is the discovery of the molecular basis of water sensing. To understand the mechanisms underlying water sensing, it is critical first to pinpoint the water-sensing niche in root tips. Studies suggest that the basal meristem and early elongation zone serve as the earliest sites of water sensing (Mehra *et al*., 2022). This zone also encompasses the oscillation zone, a key site for auxin-driven lateral root priming which is highly sensitive to water availability (De Smet *et al*., 2007; Moreno-Risueno *et al*., 2010). Future research should focus on revealing the temporal and spatial cellular resolution of root–water interaction processes to uncover the gene regulatory networks involved in the initial water perception events. This involves understanding which cell types are crucial for sensing water at the cellular scale.

With the development of high-resolution and sensitive biosensors, it has become increasingly feasible to monitor dynamically changing hormone distribution patterns in roots at the cellular scale (Balcerowicz *et al*., 2021; Rowe *et al*., 2023). Such analyses under different root adaptive responses will provide insights into how hormone redistribution regulates root–water interaction processes.

## Conclusions

### Root adaptations serve as model systems to study root–water interactions

Root adaptations such as xerobranching, hydrotropism, and hydropatterning stem from the ability of roots to sense and respond to ‘local’ water availability. As a result, these tissue-scale adaptations offer clean experimental systems with minimal systemic interference as well as enhanced temporal resolution to study molecular events—from water sensing to response. Moreover, these adaptations are conserved across various crop species (Mehra *et al*., 2023). Therefore, hormone-driven molecular mechanisms discovered through these systems provide a framework for understanding similarities and differences in how hormones regulate root–water interactions. This knowledge is vital for deciphering early responses to drought and other water-related stresses such as soil compaction (Pandey *et al*., 2021; Huang *et al*., 2022; Pandey and Bennett, 2024).

### Bridging lab models with real-world soil environments is essential for deeper insights

Most of our understanding about the molecular basis of root–water interactions comes from controlled laboratory conditions. Therefore, a significant challenge in root research is replicating soil heterogeneity in laboratory experiments to create more realistic lab-based models. Non-invasive imaging platforms such as MRI and X-ray tomography enable three-dimensional visualization of roots *in vivo* (Sturrock, 2023). However, despite this advancement, we still lack experimental platforms to study the hormone dynamics in root responses under real soil conditions. Therefore, future research should prioritize the development of innovative lab-based soil models that closely mimic natural soil environments. This will require an interdisciplinary approach that effectively integrates tools from molecular biology, soil science, and advanced imaging techniques. Innovative non-invasive visualization methods that can simultaneously capture the structural and hormone-driven molecular dynamics of roots will revolutionize our ability to study roots in their natural environment.

### Root adaptations provide opportunities to improve water stress resilience

Roots display remarkable adaptability to water availability to optimize foraging, with hormones playing a crucial role in coordinating these responses. Key responses, such as hydropatterning and xerobranching, are conserved across angiosperms, driven by similar hormone-driven molecular networks (Mehra *et al*., 2023). Studies, including those by Uga *et al.* (2011, 2013), highlight the significant influence of genotypic variability in root traits on responses to water availability. Similarly, recent research emphasizes the potential of genotypic variability in hydropatterning as a valuable framework for crop improvement (Scharwies *et al.,* 2025). Exploring this variability within species and examining the phylogenetic distribution of these root adaptive responses across diverse ecological niches could provide insights into their evolutionary significance and pave the way for potential applications for enhancing drought resilience. Understanding the fundamental mechanisms underlying these adaptations and their variability is crucial for designing root systems optimized for diverse environmental conditions. By integrating evolutionary, ecological, and molecular perspectives, these traits can be harnessed to improve water use efficiency and resilience, thereby contributing to sustainable agricultural productivity.
